# Cardiorespiratory fitness and aerobic performance adaptations to a 4-week sprint interval training in young healthy untrained females

**DOI:** 10.1007/s11332-016-0313-x

**Published:** 2016-09-14

**Authors:** Mykolas Kavaliauskas, Thomas P. Steer, John A. Babraj

**Affiliations:** 1 School of Applied Sciences, Edinburgh Napier University, 2.B.38, Sighthill Campus, Edinburgh, EH11 4BN UK; 2 Division of Sport and Exercise Sciences, Abertay University, Dundee, UK

**Keywords:** High-intensity interval training, Aerobic capacity, Cycling time trial, Critical power

## Abstract

**Purpose:**

The aim of this study was to test the effects of sprint interval training (SIT) on cardiorespiratory fitness and aerobic performance measures in young females.

**Methods:**

Eight healthy, untrained females (age 21 ± 1 years; height 165 ± 5 cm; body mass 63 ± 6 kg) completed cycling peak oxygen uptake ($\dot{V}{\text{O}}_{2}$ peak), 10-km cycling time trial (TT) and critical power (CP) tests pre- and post-SIT. SIT protocol included 4 × 30-s “all-out” cycling efforts against 7 % body mass interspersed with 4 min of active recovery performed twice per week for 4 weeks (eight sessions in total).

**Results:**

There was no significant difference in $\dot{V}{\text{O}}_{2}$ peak following SIT compared to the control period (control period: 31.7 ± 3.0 ml kg^−1^ min^−1^; post-SIT: 30.9 ± 4.5 ml kg^−1^ min^−1^; *p* > 0.05), but SIT significantly improved time to exhaustion (TTE) (control period: 710 ± 101 s; post-SIT: 798 ± 127 s; *p* = 0.00), 10-km cycling TT (control period: 1055 ± 129 s; post-SIT: 997 ± 110 s; *p* = 0.004) and CP (control period: 1.8 ± 0.3 W kg^−1^; post-SIT: 2.3 ± 0.6 W kg^−1^; *p* = 0.01).

**Conclusions:**

These results demonstrate that young untrained females are responsive to SIT as measured by TTE, 10-km cycling TT and CP tests. However, eight sessions of SIT over 4 weeks are not enough to provide sufficient training stimulus to increase $\dot{V}{\text{O}}_{2}$ peak.

## Introduction

Low-volume high-intensity interval training (HIT) is a time-efficient training paradigm involving repeated bursts of “all-out” efforts followed by a period of passive or active rest [1]. Sprint interval training (SIT), in the form of 30-s repeated Wingate tests performed 4–6 times with ~4 min of recovery, is a commonly used HIT modality [2]. SIT performed for as little as six sessions over 2 weeks has been shown to improve health and fitness in a wide range of populations [3]. Some of the reported benefits following SIT include rapid changes in aerobic energy metabolism [1], a rightward shift in the blood lactate curve during incremental cycling exercise [4] and improved anaerobic metabolism [5].

Most studies investigating SIT have used all male or mixed-sex participants [6, 7]. However, it has been suggested that females may not be as responsive to SIT as males [8]. This could be attributed to differences in the adaptive responses. For example, Esbjörnsson-Liljedahl et al. [9] reported a smaller muscle glycogen utilisation in type I fibres after a single Wingate test in females than males. Furthermore, compared to males, the glucose transporter 4 (GLUT4) levels are significantly lower in females following SIT [10]. In addition, lipid oxidation capacity as measured by the changes in the maximal activity of β-hydroxyacyl-CoA dehydrogenase (β-HAD) only increased in males after SIT [10]. Similarly, a greater mitochondrial biogenesis during SIT was reported in males compared to females [11].

Despite these sex-specific differences in skeletal muscle adaptations, Astorino et al. [12] reported similar magnitude of change in maximal oxygen uptake ($\dot{V}{\text{O}}_{{ 2\;{ \hbox{max} }}}$) and power output in a group of recreationally active men and women matched for age, physical activity and $\dot{V}{\text{O}}_{{ 2\;{ \hbox{max} }}}$ following low-volume SIT. This was further supported by Scalzo et al.’s study [11], which found no effect of sex on $\dot{V}{\text{O}}_{{ 2\;{ \hbox{max} }}}$, 40-km cycling TT and relative power output following a 3-week SIT in young, recreationally active males and females.

A retrospective study by Astorino and Schubert [13] compared changes in $\dot{V}{\text{O}}_{{ 2\;{ \hbox{max} }}}$, heart rate and fat oxidation following two types of commonly used interval training in two different groups of participants. A higher percentage of ‘non-responders’ has been found following a 2-week SIT in mixed-sex participants when compared to a 12-week high-volume, HIT in just females [13]. Specifically, 65 % of active participants showed meaningful improvement in $\dot{V}{\text{O}}_{{ 2\;{ \hbox{max} }}}$ following SIT compared to 95 % of sedentary women that completed HIT [13]. Despite methodological differences, these findings have some great practical implications for exercise prescription as SIT appears to result in a lower frequency and magnitude of adaptations related to cardiovascular fitness and metabolic health. However, the effectiveness of SIT for improving aerobic performance variables in young, untrained females remains unclear.

Therefore, this study sought to determine whether low-volume SIT, with a reduced resistance, could improve cardiorespiratory fitness and aerobic performance measures, as represented by $\dot{V}{\text{O}}_{2}$ peak, 10-km cycling TT and critical power (CP), in young, healthy, untrained females. It was hypothesised that 4 weeks of twice weekly SIT (eight sessions in total) would result in a significant improvement in all measures.

## Methods

### Participants

Eight young, healthy, untrained females, who were exercising for less than 60 min 2–3 times per week but not following a structured training programme, volunteered to participate in the study (age 21 ± 1 years; height 165 ± 5 cm; days since last menstruation—testing session 1 (pre-SIT-1): 17 ± 7 days; testing session 2 (pre-SIT-2): 22 ± 8 days; testing session 3 (post-SIT): 20 ± 9 days). Across the control and intervention periods, participants were asked to maintain their normal diet and exercise pattern, but no attempt was made to record this. Changes in body composition during the control period and post-SIT are shown in Table 1, with no statistical difference across the time points. Participants were recruited via local advertisement and provided written informed consent. Anybody who had suffered a lower limb injury in the past 6 months or with a chronic health condition was excluded from the study. The study was approved by the University Research Ethics Committee and was carried out in line with the Declaration of Helsinki.

**Table 1:** Changes in body composition during control period and post-SIT (mean ± SD)

	Control period		Post-SIT
	Pre-SIT-1	Pre-SIT-2	
Body mass (kg)	63 ± 6	62 ± 6	62 ± 7
Body fat (%)	28 ± 4	27 ± 4	27 ± 4
BMI (kg/m^2^)	23 ± 3	23 ± 2	23 ± 3

### Baseline testing

Seven days prior to baseline testing (pre-SIT-1), participants completed a familiarisation session involving a 30-s cycle maintaining between 60 and 100 r min^−1^ [14]. Participants were asked to refrain from caffeine and alcohol consumption and participation in physical activity for 24 h prior to each test [14]. All participants’ body composition wearing minimal clothing was assessed using the leg-to-leg bioelectrical impedance analysis (BIA) (Tanita TBF 300, Tanita Co., Japan) before each testing session that was separated by 48 h [15] and carried out in the following order and at the same time of day.

### Assessment of $\dot{V}{\text{O}}_{2}$ peak

Participants performed a continuous incremental cycling test to volitional exhaustion on a cycle ergometer (Monark Ergomedic 835, Monark Exercise AB, Sweden) to determine $\dot{V}{\text{O}}_{2}$ peak via an automated gas analysis system (Metalyzer^®^3B gas analyser, Cortex, Leipzig, Germany). Time to exhaustion (TTE) was also recorded using a Quantum 5500 stop clock (EA Combs Ltd., UK). Each participant completed a 5-min warm-up, maintaining a speed above 60 r min^−1^ with no applied resistance [16]. The participant then had to maintain a cadence of 70 r min^−1^ or above throughout, with 0.5 kg added to the cradle every 4 min until volitional exhaustion or they could no longer maintain the required cadence [17]. $\dot{V}{\text{O}}_{2}$ peak was defined as the highest 30-s average recorded during the test. Verbal encouragement was given throughout the test [18].

### 10-km cycling time trial

Each participant completed a 5-min warm-up, as above, prior to performing a self-paced 10-km cycling TT (Monark Ergomedic Model 835, Monark Exercise AB, Sweden). Instructions were given to each participant before each session to complete the test as fast as possible against a set resistance of 1.5 kg applied to the flywheel, ensuring that greater cadence leads to a greater power production and a faster completion time. Time was recorded following the warm-up using a Quantum 5500 stop clock (EA Combs Ltd., UK). Participants were informed of the distance covered every 1 km but not the time taken. Verbal encouragement was given throughout the test [18].

### Critical power

Participants performed a 5-min warm-up, as above, prior to the CP test. The CP test involved a 3-min “all-out” cycling effort against 4.5 % body mass [19]. The test began as soon as they reached 110 r min^−1^ and verbal encouragement was given throughout [18]. The participants were given no feedback on time, speed or power during the test. CP was calculated as the average power output over the final 30 s of the test [19].

### Control period

The participants reported back to the laboratory 4 weeks after baseline testing to repeat the tests (pre-SIT-2) in the same order with 48 h between each test. During the control period the coefficients of variation for the repeated measures were 5.2 % for $\dot{V}{\text{O}}_{2}$ peak and 8.2 % for TT.

### Sprint interval training protocol

The SIT protocol was similar to that used previously by Lindsay et al. [20]. Eight sessions of SIT were spread over a 4-week period, with two training sessions performed each week separated by at least 48 h of rest. Each session consisted of 4 × 30-s “all-out” cycling efforts performed on a Monark 894E ergometer (Varberg, Sweden) against 7 % body mass, with a 4-min recovery period between each sprint. Body mass percentages were reduced from what has been previously used due to the difference in muscle mass between males and females [21]. The weight was automatically released from the bike cradle once the participant had reached a speed of 110 r min^−1^, which initiated the start of the 30-s cycle sprint. Peak and average power output during each sprint was automatically calculated using Monark Anaerobic Test Software (version 2.24.2; Monark Exercise AB, Sweden). During the recovery period, participants remained on the bike and cycled at a low cadence (<50 r min^−1^) without a weighted resistance.

### Post-training assessment

Participants repeated baseline tests (post-SIT) 7 days after completion of the last training session, with the $\dot{V}{\text{O}}_{2}$ peak, 10-km cycling TT and CP test repeated in the same order and at the same time of the day, with 48 h between each test.

### Statistical analysis

All data are reported as mean ± standard deviation. Data were checked for skewness and kurtosis and these values did not exceed twice the standard error; therefore, the data were deemed to be normally distributed. A one-way repeated-measures ANOVA with LSD post hoc testing was used to compare between the three time points. The assumption of sphericity was determined by Mauchly’s test. Significance was accepted at *p* < 0.05 in all analyses. Cohen’s *d* effect sizes were calculated using the correction for dependence between means [22] and defined as follows: *d* <0.2 trivial effect; *d* = 0.2–0.5 small effect; *d* = 0.6–1.1 moderate effect; *d* >1.2 large effect.

## Results

### $\dot{V}{\text{O}}_{2}$ peak

**Fig. 1:**
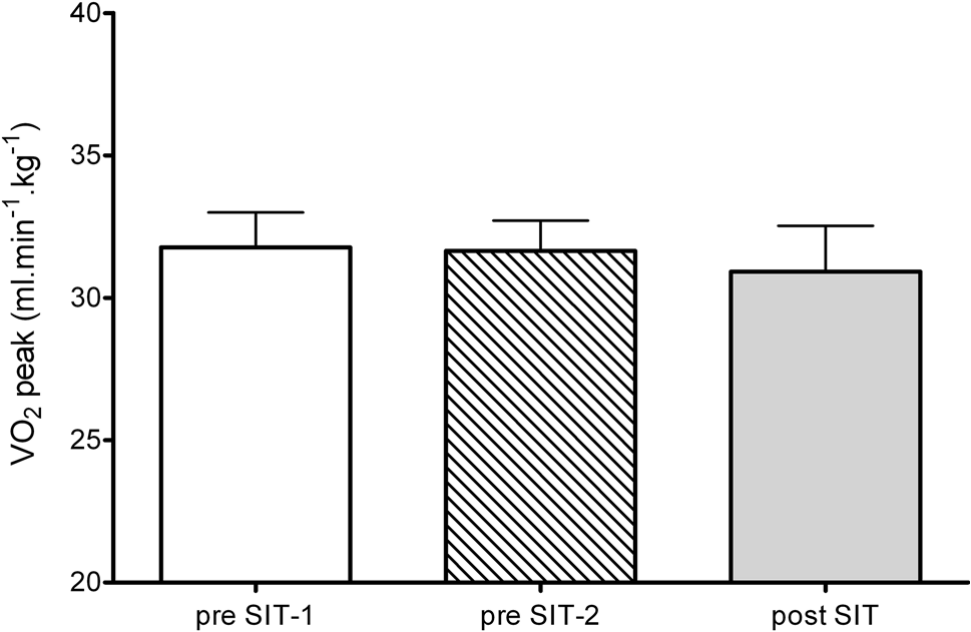
Absolute changes in $\dot{V}{\text{O}}_{2}$ peak during control period (pre-SIT-1 and pre-SIT-2) and post-SIT

$\dot{V}{\text{O}}_{2}$ peak was not significantly different during the control period (pre-SIT-1: 31.8 ± 3.5 ml kg^−1^ min^−1^; pre-SIT-2: 31.7 ± 3.0 ml kg^−1^ min^−1^; *p* > 0.05; Fig. 1). Following 4 weeks of SIT, $\dot{V}{\text{O}}_{2}$ peak was not significantly different compared to pre-training (pre-SIT-2: 31.7 ± 3.0 ml kg^−1^ min^−1^; post-SIT: 30.9 ± 4.5 ml kg^−1^ min^−1^; *p* > 0.05; Fig. 1).

### Time to exhaustion

**Fig. 2:**
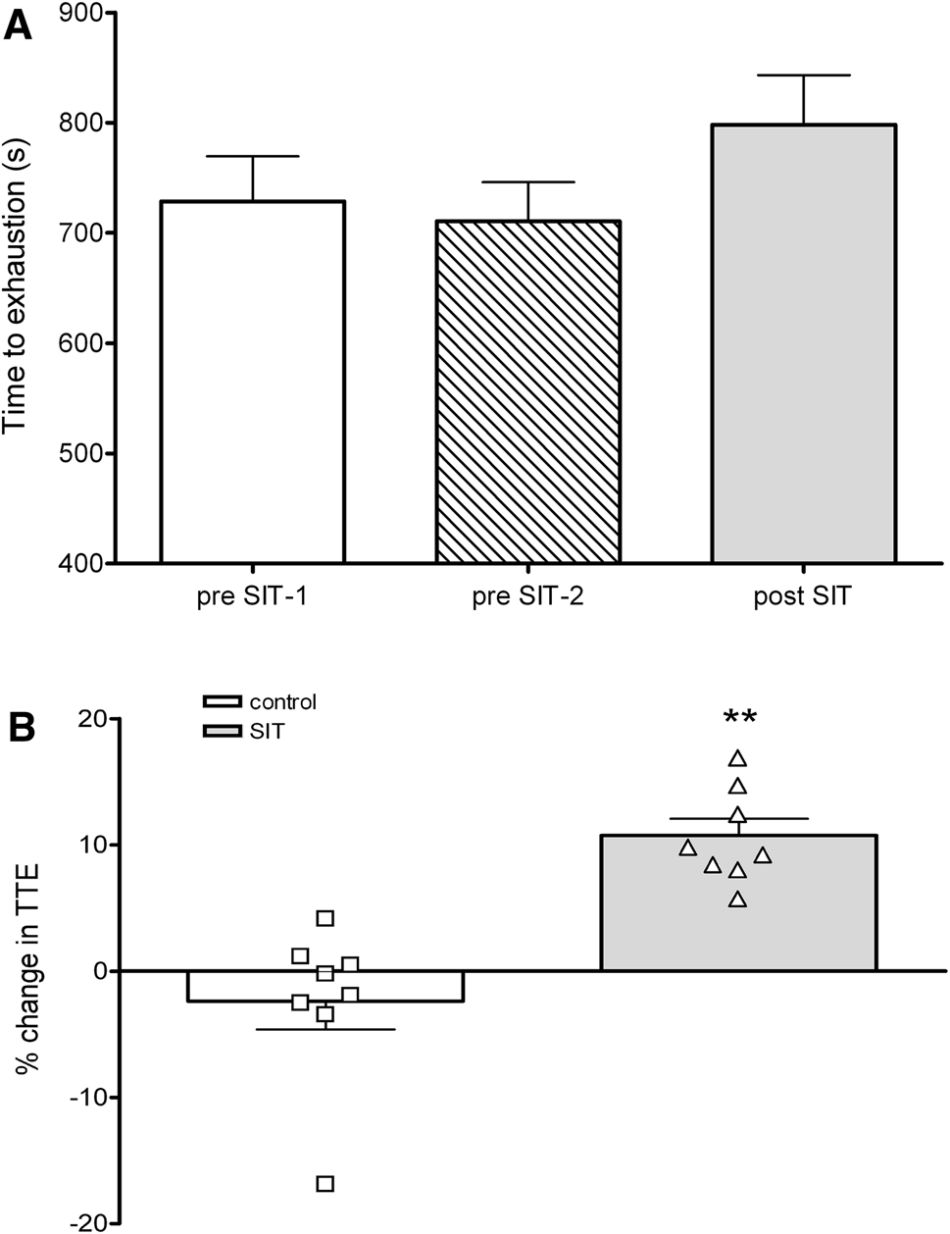
**a** Absolute changes in time to exhaustion during control period (pre-SIT-1 and pre-SIT-2) and post-SIT. **b** Mean and individual percentage changes in time to exhaustion during control period and after 4 weeks of SIT training. ^†^ *p* < 0.005 pre-SIT2 compared to post-SIT; **p < 0.005 change over control period compared to change over SIT period

TTE performance was not significantly different during the control period (pre-SIT-1: 728 ± 117 s; pre-SIT-2: 710 ± 101 s; *p* > 0.05; Fig. 2a). Following 4 weeks of SIT, TTE performance was significantly improved by 12 ± 4 % (pre-SIT-2: 710 ± 101 s; post-SIT: 798 ± 127 s; *p* = 0.001; *d* = −2.6; Fig. 2a). The magnitude of change was significantly greater following SIT compared to the control period (pre-SIT: −2 ± 6 %, post-SIT: 12 ± 4 %; *p* = 0.004; *d* = −0.9; Fig. 2b).

### 10-km cycling time trial

**Fig. 3:**
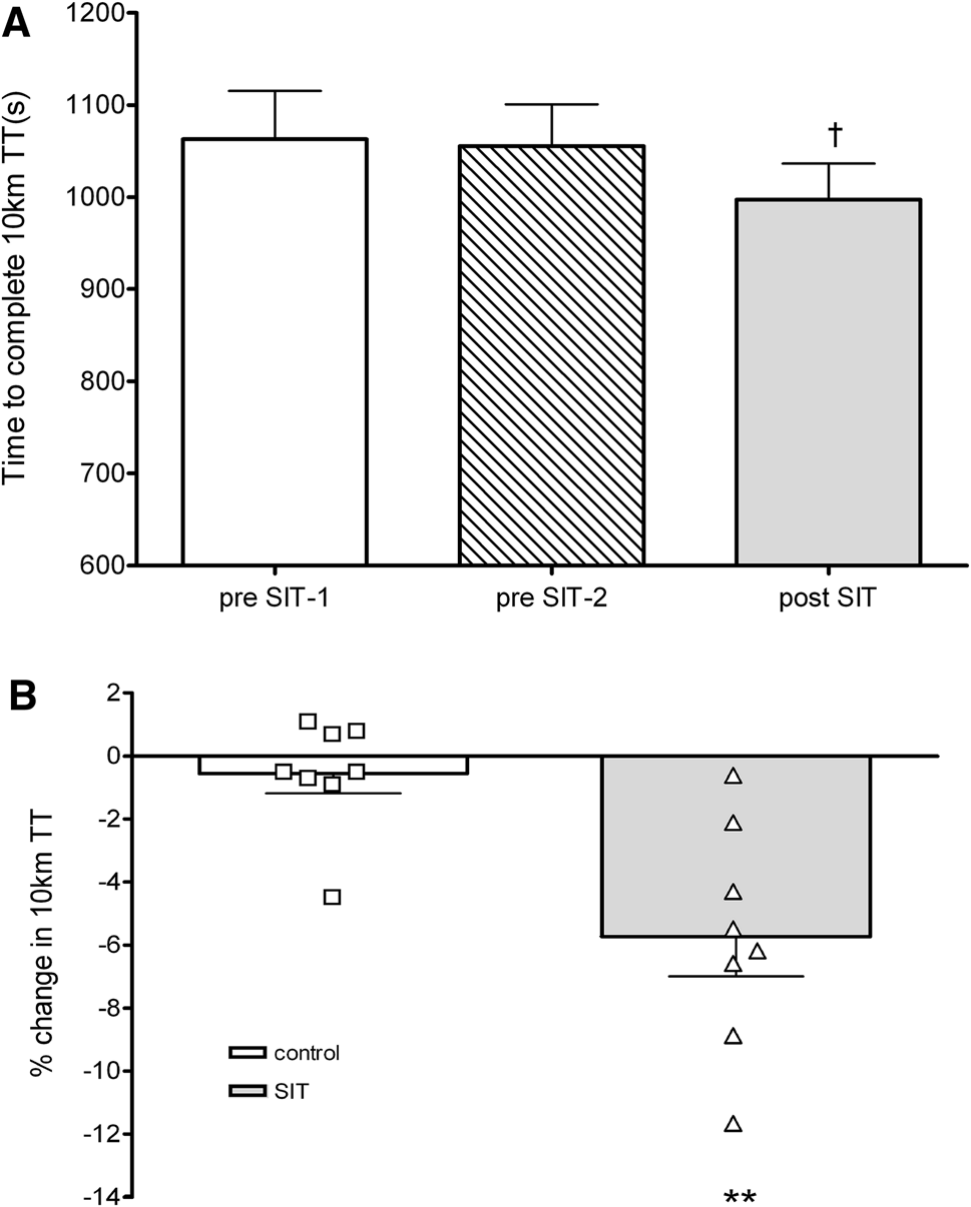
**a** Absolute changes in 10-km cycling time trial performance during control period (pre-SIT-1 and pre-SIT-2) and post-SIT. **b** Mean and individual percentage changes in 10-km cycling time trial performance during control period and after 4 weeks of SIT training. ^†^ *p* < 0.005 pre-SIT2 compared to post-SIT; **p < 0.005 change over control period compared to change over SIT period

Time trial performance was not significantly different during the control period (pre-SIT-1: 1063 ± 149 s; pre-SIT-2: 1055 ± 129 s; *p* > 0.05; Fig. 3a). Following 4 weeks of SIT, TT performance was significantly reduced by 6 ± 4 % (pre-SIT-2: 1055 ± 129 s; post-SIT: 997 ± 110 s; *p* = 0.004; *d* = 1.7; Fig. 3a). The magnitude change was significantly greater following SIT compared to the control period (pre-SIT: −1 ± 2 %; post-SIT: −6 ± 4 %; *p* = 0.002; *d* = 1.3; Fig. 3b).

### Critical power

**Fig. 4:**
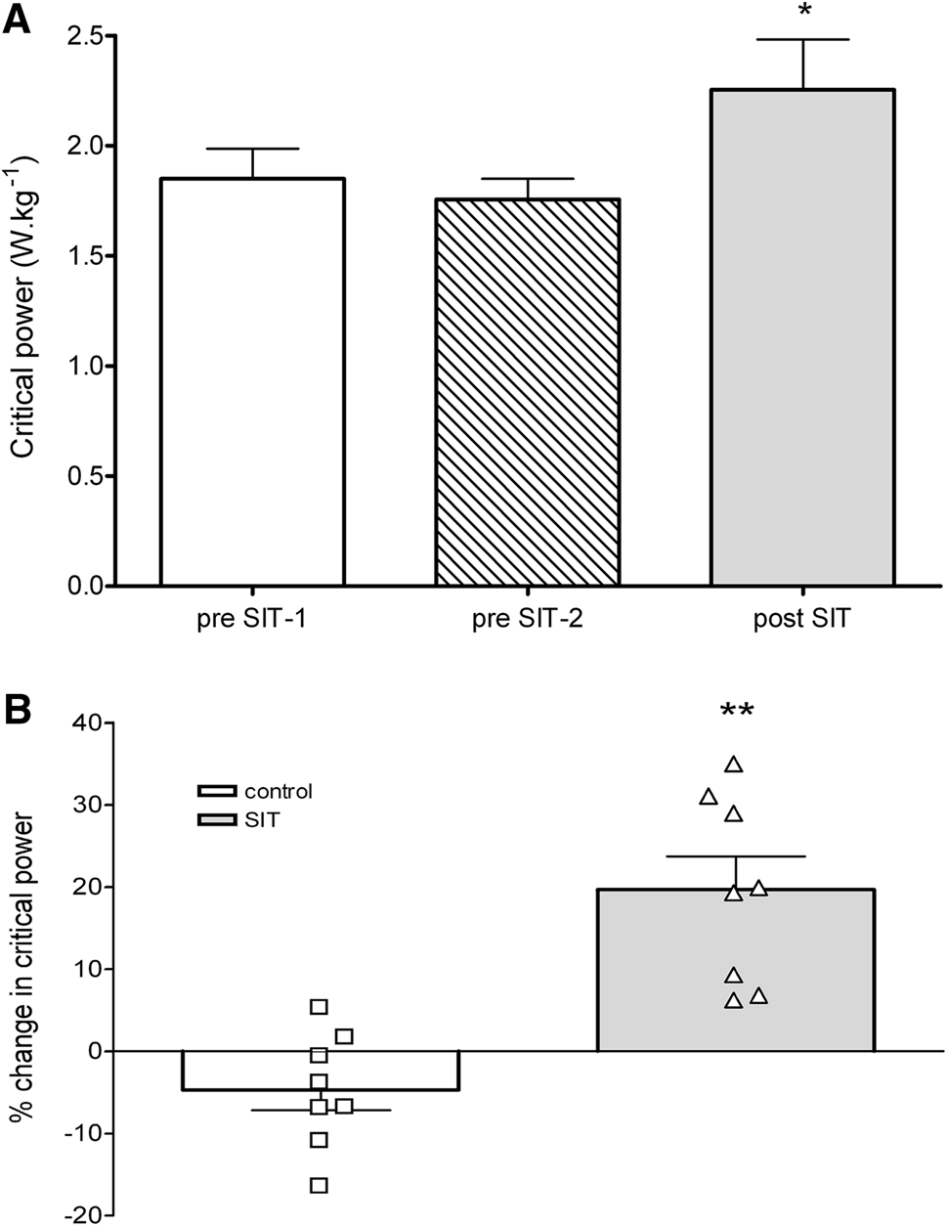
**a** Absolute changes in critical power during control period (pre-SIT-1 and pre-SIT-2) and post-SIT. **b** Mean and individual percentage changes in critical power during control period and after 4 weeks of SIT training. *p < 0.01 pre-SIT2 compared to post-SIT; **p < 0.005 change over control period compared to change over SIT period

CP was not significantly different during the control period (pre-SIT-1: 1.9 ± 0.4 W kg^−1^; pre-SIT-2: 1.8 ± 0.3 W kg^−1^; *p* > 0.05; Fig. 4a). Following 4 weeks of SIT, CP was significantly increased by 27 ± 18 % (pre-SIT-2: 1.8 ± 0.3 W kg^−1^; post-SIT: 2.3 ± 0.6 W kg^−1^; *p* = 0.01; *d* = −2.5; Fig. 4a). The magnitude of change was significantly higher following SIT compared to the control period (pre-SIT: −4 ± 6 %, post-SIT: 27 ± 18 %; *p* = 0.002; *d* = −0.9; Fig. 4b).

### Power production during training

Peak power was significantly greater following 4 weeks of SIT (training session 1: 575 ± 85 W; training session 8: 617 ± 76 W; *p* = 0.04; *d* = −0.9). However, average power was not significantly different following SIT (training session 1: 399 ± 52 W; training session 8: 413 ± 42 W; *p* = 0.15; *d* = −0.6). The sum of the peak power across all four cycling sprints was significantly greater following SIT (training session 1: 2024 ± 271 W; training session 8: 2213 ± 269 W; *p* = 0.01; *d* = −1.3). The sum of the average power across all four cycling sprints was also significantly greater following SIT (training session 1: 1406 ± 153 W; training session 8: 1478 ± 153 W; *p* = 0.01; *d* = −1.3).

## Discussion

The present study showed that a 4-week SIT programme consisting of 4 × 30-s “all-out” sprints performed twice per week improves aerobic performance measures in young, untrained females. A time-efficient SIT protocol significantly improved TTE, 10-km cycling TT and CP. Based on individual results, all participants (*n* = 8) were responsive to SIT by showing positive change in these endurance parameters, but there was a variable level of responses. In addition, eight sessions of SIT, at a reduced resistance, did not provide sufficient training stimulus to improve cardiorespiratory fitness as measured by $\dot{V}{\text{O}}_{2}$ peak.

Following eight sessions of SIT over 4 weeks, we report no improvements in $\dot{V}{\text{O}}_{2}$ peak in females (Fig. 1). The non-significant change in $\dot{V}{\text{O}}_{2}$ peak in the current study is similar to the findings by Burgomaster et al. [6] who found no changes in $\dot{V}{\text{O}}_{2}$ peak in eight healthy participants, including two females, following 2 weeks of SIT. In contrast, a meta-analysis by Gist et al. [2] reported ~8 % increase in the maximal oxygen uptake ($\dot{V}{\text{O}}_{{ 2\;{ \hbox{max} }}}$) following SIT in young, healthy participants in comparison to no-exercise control groups. When considering sex-specific responses, the mean effects of HIT on $\dot{V}{\text{O}}_{{ 2\;{ \hbox{max} }}}$ in active, non-athletic females were shown to be 3.6 % [23]. Scalzo et al. [11] found an even greater improvement (7.6 %) in $\dot{V}{\text{O}}_{{ 2\;{ \hbox{max} }}}$ in young, recreationally active females following a 3-week SIT intervention consisting of three sessions per week.

Training variables, namely training frequency and total length of the SIT programme used in this study may explain the non-significant change in $\dot{V}{\text{O}}_{2}$ peak. Incidence of ‘non-responders’ for $\dot{V}{\text{O}}_{{ 2\;{ \hbox{max} }}}$ following 2 weeks of SIT was reported to be 35 % [13], whereas based on five previous SIT studies lasting between 3 and 6 weeks the incidence of non-response for $\dot{V}{\text{O}}_{2}$ peak has been shown to be 22 % [24]. This suggests that shorter SIT programmes are associated with a higher percentage of ‘non-responders’. In addition, there is a higher magnitude of change in $\dot{V}{\text{O}}_{{ 2\;{ \hbox{max} }}}$ following longer SIT programmes. This has been recently demonstrated by Bagley et al. [25] who found that females improved $\dot{V}{\text{O}}_{{ 2\;{ \hbox{max} }}}$ by 19 % following a 12-week SIT intervention consisting of 4 bouts of 20-s ‘maximal efforts’ performed three times per week. Interestingly, females were also more responsive to SIT than men as demonstrated by greater $\dot{V}{\text{O}}_{{ 2\;{ \hbox{max} }}}$ gains [25].

Training frequency appears to be another important variable determining the physiological adaptations to SIT programmes. No ‘non-responders’ were reported when participants trained four times per week in comparison to a relatively high number of ‘non-responders’ (37 %) when training was performed three times per week [24]. Therefore, training frequency should be considered when designing SIT programmes as two sessions per week are not enough to reach a minimum training dose needed to increase $\dot{V}{\text{O}}_{2}$ peak in young, untrained females.

Despite no changes in $\dot{V}{\text{O}}_{2}$ peak, TTE during the incremental cycling test was significantly increased by 12 ± 4 % following SIT compared to the control period (Fig. 2a, b). In recreationally active participants, exercise TTE when cycling at ~80 % $\dot{V}{\text{O}}_{2}$ peak increased by 100 % following six sessions of SIT over 2 weeks [6]. In contrast, trained male participants increased TTE by 4 % after six sessions of SIT [4]. Improvements in TTE may be explained through increase in mitochondrial enzyme content and activity [6] and a rightward shift in the blood lactate curve [4] after SIT. Although, these mechanisms were not measured it seems reasonable to suggest similar adaptations occurring in the female participants in this study.

Together with an improved endurance capacity (i.e. TTE), there was a significant reduction (6 ± 4 %) in self-paced 10-km cycling TT following SIT compared to the control period (Fig. 3a, b). This is similar to the findings in male participants as six sessions of SIT have been shown to increase cycling TT performance by between 6 and 10 % [4, 26]. With no improvement in $\dot{V}{\text{O}}_{2}$ peak reported, it would suggest that the improvement in TT performance was due to peripheral adaptations. Indeed, with the TT performed against the same resistance, it suggests a greater ability to maintain a higher cadence after SIT. This ability to maintain a higher cadence may be related to improved oxygen kinetics. As cycling cadence increases there is an increase in oxygen consumption, which is lower with training due to improved oxygen extraction [27]. Future work should test the changes in oxygen kinetics and its effect on TT performance following SIT in females.

We also demonstrate, for the first time, that one of the endurance adaptations to SIT is an increase in CP, which improved by 27 ± 18 % after eight sessions of SIT (Fig. 4a, b). CP could be viewed as the highest sustainable work rate [28]. Following 12 HIT sessions over 4 weeks, CP has been shown to be increased by approximately 10 % [29]. However, changes in CP have not been reported following SIT. A study by Demarle et al. [30] demonstrated that after 16 sessions of long-duration HIT the oxygen deficit of an exercise bout is reduced. Furthermore, following six sessions of SIT over 2 weeks in young, healthy participants, there is an enhanced oxygen extraction rate within skeletal muscle [31]. It is predicted that increasing the rate of oxygen extraction requires increased mitochondrial volume [32]. It has been well documented that SIT increases both mitochondrial enzyme content and activity in the skeletal muscle [1, 6]. Therefore, similar adaptations in the present study would be expected to increase the capacity for muscle fractional oxygen extraction, leading to an increased CP.

## Limitations

Our study is not free of limitations. First, dietary intake and physical activity outside the training intervention were not monitored. However, no changes in body composition (Table 1) throughout the study suggests that participants’ normal dietary practices and physical activity patterns remained unchanged. Post-training assessments were performed 7 days after the final training session, which is in contrast to other studies that examined training-induced changes within 48–96 h of the final session [6, 7]. This was done to help determine whether adaptations following SIT can be maintained for at least a week. In addition, we did not compare the effects of SIT to other types of training, such as traditional endurance training and HIT, as done in previous studies [13, 33]. It should be noted, though, that studies have failed to fully control exercise workload as no studies to date have matched for increase in daily energy expenditure following each type of exercise. Finally, the current study provides information on cardiorespiratory fitness and aerobic performance adaptations to SIT in a small female population (*n* = 8). However, it has been shown that following endurance training there is a wide range of adaptations to $\dot{V}{\text{O}}_{{ 2\;{ \hbox{max} }}}$ [34] and this can be seen in our data as well (Fig. 1). Therefore, there is a need for larger population-based studies to determine the distribution of adaptation and possible gene/training interactions with SIT.

## Conclusion

The findings of the current study show that SIT can be used as a time-efficient method to improve aerobic performance measures in young, untrained females. In terms of practical relevance, female student-athletes, who only train recreationally and do not necessarily follow a structured training programme may benefit from doing SIT. However, to optimise SIT prescription, training frequency and total length of the SIT programme should be considered, particularly if the main goal is to increase cardiorespiratory fitness.
